# Methods on LDL particle isolation, characterization, and component fractionation for the development of novel specific oxidized LDL status markers for atherosclerotic disease risk assessment

**DOI:** 10.3389/fmed.2022.1078492

**Published:** 2023-01-05

**Authors:** Polyxeni Papadea, Marianna Skipitari, Electra Kalaitzopoulou, Athina Varemmenou, Maria Spiliopoulou, Marios Papasotiriou, Evangelos Papachristou, Dimitrios Goumenos, Anny Onoufriou, Eleftheria Rosmaraki, Irene Margiolaki, Christos D. Georgiou

**Affiliations:** ^1^Department of Biology, University of Patras, Patras, Greece; ^2^Department of Medicine, University of Patras, Patras, Greece; ^3^Department of Nephrology, General University Hospital of Patras, Patras, Greece; ^4^Department of Microbiology, General University Hospital of Patras, University of Patras Medical School, Patras, Greece

**Keywords:** oxidized LDL, apoB100, LDL lipid fractions, LDL-C, HDL-C, clinical markers, atherosclerosis, cardiovascular diseases

## Abstract

The present study uses simple, innovative methods to isolate, characterize and fractionate LDL in its main components for the study of specific oxidations on them that characterize oxidized low-density lipoprotein (oxLDL) status, as it causatively relates to atherosclerosis-associated cardiovascular disease (CVD) risk assessment. These methods are: (a) A simple, relatively time-short, low cost protocol for LDL isolation, to avoid shortcomings of the currently employed ultracentrifugation and affinity chromatography methodologies. (b) LDL purity verification by apoB100 SDS-PAGE analysis and by LDL particle size determination; the latter and its serum concentration are determined in the present study by a simple method more clinically feasible as marker of CVD risk assessment than nuclear magnetic resonance. (c) A protocol for LDL fractionation, for the first time, into its main protein/lipid components (apoB100, phospholipids, triglycerides, free cholesterol, and cholesteryl esters), as well as into LDL carotenoid/tocopherol content. (d) Protocols for the measurement, for the first time, of indicative specific LDL component oxidative modifications (cholesteryl ester-OOH, triglyceride-OOH, free cholesterol-OOH, phospholipid-OOH, apoB100-MDA, and apoB100-DiTyr) out of the many (known/unknown/under development) that collectively define oxLDL status, which contrasts with the current non-specific oxLDL status evaluation methods. The indicative oxLDL status markers, selected in the present study on the basis of expressing early oxidative stress-induced oxidative effects on LDL, are studied for the first time on patients with end stage kidney disease on maintenance hemodialysis, selected as an indicative model for atherosclerosis associated diseases. Isolating LDL and fractionating its protein and main lipid components, as well as its antioxidant arsenal comprised of carotenoids and tocopherols, paves the way for future studies to investigate all possible oxidative modifications responsible for turning LDL to oxLDL in association to their possible escaping from LDL’s internal antioxidant defense. This can lead to studies to identify those oxidative modifications of oxLDL (after their artificial generation on LDL), which are recognized by macrophages and convert them to foam cells, known to be responsible for the formation of atherosclerotic plaques that lead to the various CVDs.

## 1. Introduction

Atherosclerosis is a highly prevalent disease worldwide that accounts as the underlying cause in over 50% of the deaths in the western societies (1). Some of its main risk factors are high LDL-cholesterol (LDL-C) levels, oxidative stress (OS) (2), elevated levels of inflammatory markers (3), high blood pressure (4), diabetes (3), menopause (5, 6), obesity (7), family history, genetic factors, and an unhealthy diet (8). Atherosclerosis is known to lead to arterial obstruction and cardiovascular diseases (CVDs) *via* the narrowing of arterial lumen, due to the formation of atherosclerotic lesions in the arterial walls (9). The observed atherosclerotic lesions are known to be caused by accumulation of oxidized low-density lipoproteins (oxLDLs) in the subendothelial arterial space and their impending continuous phagocytosis from existing macrophages, which then undergo apoptosis and are turned into foam cells (10). Nonetheless, LDL-C is the traditional clinical marker thus far used in assessing CVDs risk development (11). However, numerous studies have pointed out the inefficacy of LDL-C in predicting CVDs risk (12–17), as it is also shown by cases where even subjects with normal LDL-C levels manifest cardiovascular events, even after statin treatment (11, 12, 14, 16). Moreover, atherosclerosis shows great variability in its clinical expression at each LDL-C level (17).

Despite the proven central causative role of oxLDL in atherosclerosis development (18), it has been only used in clinical studies as a prognostic marker for CVDs risk assessment but with contradictory results, possibly because it is being assessed non-specifically as a whole particle (16, 18–23). This is mainly due to the lack of methods (a) for determination of specific oxidative modifications in the main protein/lipid components of oxLDL particles, and (b) for LDL fractionation into its main components. The latter is exacerbated by the fact that the current LDL isolation methods are clinically impractical, as being based on time consuming, cumbersome and instrumentation costly ultracentrifugation (24, 25) and affinity chromatography methods (26).

**FIGURE 1:**
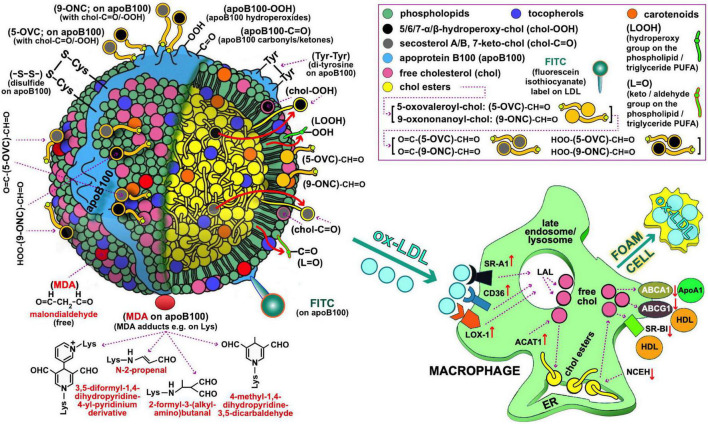
Oxidized LDL as depicted by possible oxidative modifications of its main components, possibly recognized by macrophages (**left** and **right** panel, respectively) for oxLDL uptake during the course of atherosclerosis development. Starting with the oxidative modifications of the LDL lipid components, clinically important are those of cholesterol (free or esterified) and particularly the following: (i) Those carrying keto-groups (-C=O; i.e., in 7-keto-chol, and secosterol-B and -A with 1 and 2 keto-groups, respectively), to be quantified by a method under development by our lab. (ii) Those having a hydroperoxy group (HOO-; i.e., 5α-HOO-chol, 5β-HOO-chol, 6α-HOO-chol, and 6β-HOO-chol) (85); there is also the 7-HOO-chol, which is converted to the 7-keto-chol (86). Oxidatively modified cholesterol can also exist in its ester form, and is expected to translocate to the surface of the LDL particle as being polar (shown with large red arrows). Moreover, 5-oxovaleroyl-chol (5-OVC) and 9-oxononanoyl-chol (9-ONC) represent important oxidative modifications on the polyunsaturated fatty acid (PUFA) part of the cholesterol esters (87), which are also expected to translocate to the LDL’s surface due to their polar aldehyde end group [see (5-OVC/9-ONC)-CH=O], as will also translocate their O=C-/HOO-chol modifications [see O=C-/HOO-(5-OVC/9-ONC)-CH=O]. Additionally, these oxidative modifications of 5-OVC and 9-ONC can cross-link with apoB100 due to their very reactive aldehyde end (87). All these aldehydic product can be collectively quantified by a method under development by our lab. PUFA can be also oxidized in lipid hydroperoxides and keto/aldehydes (LOOH and L=O, respectively), and when present in the LDL phospholipids/triglycerides are expected to translocate to the LDL lipid monolayer surface due to their polarity (depicted by the small red arrows); LOOH can further oxidize to malondialdehyde (MDA), which could accumulate on the LDL surface as being polar (see depicted as red circles), or react with certain amino acids (88) in apoB100 (depicted as red oval). L=O can be quantified by a method under development by our lab. Extending to apoB100 oxidative modifications, of particular interest are the following: the OS-induced formation of dityrosines (89), and disulfides (between two Cys; -S-S-) (90), hydroperoxides (apoB100-OOH) (50), and carbonyls (apoB100-C=O) (91, 92), the last three by methods developed by our lab. As illustrated in **(right)** panel, macrophages recognize and bind oxLDL with several scavenger receptors (SR, e.g., SR-A1, CD36, and LOX-1). Future studies, could address quantitatively oxLDL uptake by macrophages by flow cytometry in order to be used as an *ex vivo* clinical marker for CVD risk assessment and for studies to identify the oxidative modifications on oxLDL recognized by macrophages. In such studies, oxLDL from patients with diseases prone to atherosclerosis development, and LDL from healthy subjects to be used either as control or artificially turned to oxLDL with known oxidative modifications, can be labeled with, e.g., fluorescein isothiocyanate [FITC; preliminarily achieved by our lab as described elsewhere (93, 94)]. Under normal (healthy) conditions of low OS, lysosomal acid lipase (LAL) in late endosomes/lysosomes, degrades LDL cholesteryl esters (CE) to free cholesterol (chol) and fatty acids, while acyl coenzyme A:cholesterol acyltransferase-1 (ACAT1), contributes to formation of CE from the chol in the endoplasmic reticulum (ER) where they accumulate. Neutral CE hydrolase (NCEH) converts CE to chol that is transported outside the cells *via* the ATP-binding cassette (ABC) transporters ABCA1 and ABCG1, and also SR-BI, with the latter two passing chol to HDL *via* apolipoprotein A-1 (ApoA-1), thereby ensuring cholesterol homeostasis control. In high OS-promoted atherosclerosis, this control is deregulated, leading to increased SR expression and subsequent oxLDL elevated uptake. In contrast, expression of the chol transporters ABCA1 and ABCG1 is suppressed, which diminishes cholesterol efflux and promotes its deposition in macrophages. At the same time, ACAT1 and NCEH are upregulated and downregulated, respectively, which leads to accumulation of CE. Concurrent operation of these mechanisms results in excessive deposition of lipids and transformation of macrophages to foam cells, as also outlined elsewhere (95). LDL and macrophage draws are major modifications from Berg et al. (96).

The present study introduces innovative methodologies for the isolation of LDL, its purity assessment and particle characterization, and its fractionation into its main protein and lipid components apoB100, phospholipids, triglycerides, free cholesterol, and cholesteryl esters. These are then studied to identify, for the first time, certain early-OS-generated specific oxidative modifications on them (out of the many possibly existing due to the multifactorial and disease-/patient-differentiated manifestation of OS, Figure 1), in order to be evaluated as new potential clinical markers for CVDs risk assessment. These markers are applied indicatively on atherosclerosis-prone (27–29) end stage renal disease (ESRD) on maintenance hemodialysis (CKD-5d) patients, suffering from a disease where CVDs are the most common and severe co-morbidities in over 50% of CKD-5d patients (30), as well as the main cause of death (30, 31). A wider criterion for CKD-5d study group selection is the fact that those patients exhibit high OS (28).

## 2. Materials and equipment

### 2.1. Instrumentation

Balance (Kern, model 770/65/6J)
Bench top centrifuge (Hermle, model Z206A)
Centrifugal vacuum concentrator (CHRIST, model RVC 2-18), connected to a vacuum pump (KNF, model N 820.3 FT.18)
Corex glass tubes, 15 ml
Drying and heating chamber (BINDER, model E 28), or any oven with temperature regulation
Glass Pasteur pipettes (i.d. 0.5 cm, 22 cm length, by Hirschmann Laborgeräte GmbH & Co., Germany)
Magnetic stirrer (FALC, model F30)
Microcentrifuge clear tubes, 1.5 and 2 ml (VWR, cat. no. 89000-028)
Micropipettes (adjustable volume) 2.5 μl, 10 μl, 20 μl, 100 μl, 200 μl, 1 ml, and tips (Eppendorf Research)
Microcuvette for absorbance measurements (12.5 Å × 12.5 Å × 45 mm external dimensions, 4 mm internal window and 9 mm bottom, 1.16 ml, quartz; Starna 9/B/9/Q/10)
Microcuvette for fluorescence measurements (45 × 4 mm, 0.5 ml, quartz; Starna SOG/Q), fitted in a Starna, FCA 4 adapter
Mini-PROTEAN 3 cell for protein electrophoresis (Bio-Rad), connected to a high-current power supply (PowerPac HC of 250 V, 3.0 A, 300 W output, Bio-Rad)
pH meter (Metrohm, model 827 pH lab)
Refrigerated microcentrifuge (Eppendorf Research, model 5417R)
Semi-micro analytical balance (Shimadzu, model AUW120D, 120 G/0.1 MG)
Spectrofluorometer (Shimadzu, model RF-1501)
Spectrophotometer (Hitachi, model UV–VIS U-1800)
Table top refrigerated centrifuge (Henle, model Z 36 HK)
TLC aluminum sheets silica gel 60 (without fluorescent indicator) pre-coated 25 sheets 20 × 20 cm, layer thickness 0.2 mm (Merck, cat. no. 08808398)
UV-C hand lamp, wavelength: 312 and 254 nm (Vilber lourmat, model VL-6MC)
Vortex (FALC, model MIX 10)
Waterbath (Memmert, model W270).

### 2.2. Reagents

Acetone (AC; Merck, cat. no. 01-6300117)
Acetic acid, glacial (Sigma, cat. no. 537020)
Acrylamide (>99%; Bio-Rad, cat. no. 161-0101)
Ammonium ferrous sulfate hexahydrate (Sigma, cat. no. 203505)
Ammonium persulfate (Sigma, cat. no. A3678)
ApoB100 (Sigma, cat. no. SRP6302)
Benzene (99.5%; Chem-Lab, cat. no. CL00.0215.1000)
Bromophenol blue (Sigma, cat. no. B5525)
1-Butanol (ButOH; Chem-Lab, cat. no. CL00.0220)
Butylated hydroxyanisole (BHA; Sigma, cat. no. B1253)
Butylated hydroxytoluene (BHT; Sigma, cat. no. W218405)
Calcium chloride granular (CaCl_2_; Merck, cat. no. C1016)
Chloroform (CHCl_3_; Merck, cat. no. 1.02445)
Cholesterol (>95%; TCI, cat. no. C0318)
Coomassie Brilliant Blue G-250 (CBB G-250; Serva, cat. no. C.I.42655)
Diethyl ether (Merck, cat. no. 100921)
Ethanol (EtOH; Merck, cat. no. 159010); caution, highly flammable
Ethyl acetate (EA; Sigma, cat. no. 270989)
Glycerol (Sigma, cat. no. G5516)
Glycine (Sigma, cat. no. G6388)
Heparin 5000 i.u./ml – 5 ml (25,000 i.u.) (LEO, cat. no. 013236-00)
Hexane (Merck, cat. no. 104374)
Hydrochloric acid (HCl ≥37% w/w; Fluka, cat. no. 84415)
7-Keto-cholesterol (Avanti Pollar Lipids, cat. no. 700015P)
LDL-cholesterol kit (LDL-C kit; Medicon, cat. no. 1418-0227)
Magnesium chloride hexahydrate (MgCl_2_6 H_2_O; Merck, cat. no. 102367)
2-Mercaptoethanol (Sigma, cat. no. M6250)
Methanol (100%) for HPLC (Sigma-Aldrich, cat. no. 34860)
N-(2-hydroxyethyl)piperazine-N′-2-ethane sulfonic acid (HEPES; C_8_H_18_N_2_O_4_S; Serva, cat. no. 25245)
N,N′-methylene-bis-acrylamide (Merck, cat. no. 2610-OP)
Octane (-iso) (Isooctane; Chem-Lab, cat. no. CL00. 1512)
Polyethylene glycol 6000 (PEG-6000; Serva, cat. no. 33137)
Rhodamine 6G (Sigma, cat. no. R4127)
Silica gel high-purity grade, pore size 60 Å, 70–230 mesh, 63–200 μm, for column chromatography (Fluka, cat. no. 60741-1KG)
Sodium chloride (NaCl; Sigma, cat. no. 433209)
Sodium citrate (C_6_H_5_Na_3_O_7_2H_2_O; Sigma, cat. no. W302600)
Sodium dodecyl sulfate (SDS; Bio-Rad, cat. no. 1610302)
Sodium hydroxide (NaOH; Merck, cat. no. 106462)
Sodium (tri-)phosphate dodecahydrate (N a_3_PO_4_⋅12H_2_O; Merck, cat. no. 106578)
Sulfuric acid (95–97%; Merck, cat. no. 100731)
TEMED (Sigma, cat. no. T22500)
2-Thiobarbituric acid (TBA; Serva, cat. no. 36108.01)
Trichloroacetic acid (TCA; Merck, cat. no. 1008070100)
Tris-base (MP Biomedicals, cat. no. 02103133)
Tris hydrochloride (Merck, cat. no. 10812846001)
Xylenol orange tetrasodium salt (XO; Alfa Aesar, cat. no. 41379).

### 2.3. Reagent standard solutions

• *64 mM Citrate buffer, pH 5.12:* For 200 ml, dissolve (in ddH_2_O) 3.764 g anhydrous Na-citrate, and adjust pH with HCl. Before use adjust pH to exactly 5.12 at RT.
• *Heparin stock:* 5,000 U ml^–1^.
• *Hepes-NaCl-MgCl_2_-CaCl_2_, pH 7.2 (Hepes solution):* For 20 ml, dissolve (in ddH_2_O) 0.026 g Hepes (final 5 mM), 0.023 g NaCl (final 20 mM), 0.0080 g MgCl_2_⋅6H_2_O (final 2 mM), and 0.0088 g anhydrous CaCl_2_ (final 4 mM), and adjust pH with HCl.
• *4% NaCl:* For 5 ml, dissolve (in ddH_2_O) 0.2 g NaCl.
• *20 mM Tris buffer, pH 7.7:* For 100 ml, dissolve (in ddH_2_O) 0.242 g Tris-base and adjust pH with HCl.
• *5 M MgCl_2_:* For 7.5 ml, dissolve 7.625 g MgCl_2_⋅6H_2_O in ∼2.25 ml ddH_2_O at 37°C, and adjust volume up to 7.5 ml.
• *400/400 mM BHA/BHT:* For 4 ml, dissolve in 3.39 ml EtOH, 0.288 g BHA and 0.353 g BHT.
• *50/50 mM BHA/BHT:* For 2 ml, dissolve in 2 ml EtOH, 0.018 g BHA and 0.022 g BHT.
• *50 mM Phosphate (Pi) buffer, pH 7.4:* For 20 ml, dissolve (in ddH_2_O) 0.38 g Na_3_PO_4_⋅12H_2_O and adjust pH with HCl.
• *CHCl_3_:MetOH 3:1 (v/v):* For 16 ml, mix 12 ml CHCl_3_ with 4 ml MetOH.
• *80:1 Isooctane:EA (v/v) mixture:* For 81 ml, mix 80 ml isooctane with 1 ml ethyl acetate.
• *20:1 Isooctane:EA (v/v) mixture:* For 21 ml, mix 20 ml isooctane with 1 ml ethyl acetate.
• *75:25 Isooctane:EA (v/v) mixture:* For 10 ml, mix 7.5 ml isooctane with 2.5 ml ethyl acetate.
• *50:50:1 Hexane:diethyl ether:acetic acid (v/v/v):* For 11 ml, mix 5 ml hexane, 5 ml diethyl ether and 1 ml acetic acid.
• *0.005% Rhodamine 6G:* For 100 ml, dissolve (in ddH_2_O) 0.005 g Rhodamine 6G.
• *2.5% Diethyl ether:* For 6 ml, mix 5.85 ml benzene with 0.15 ml diethyl ether.
• *30% Acrylamide:* For 100 ml, dissolve (in ddH_2_O) 29.2 g acrylamide and 0.8 g bis-acrylamide.
• *10% Ammonium persulfate:* For 10 ml, dissolve (in ddH_2_O) 1 g of ammonium persulfate.
• *10% SDS:* For 10 ml, dissolve (in ddH_2_O) 1 g SDS.
• *1.5 M Tris–HCl buffer, pH 8.8:* For 250 ml, dissolve (in ddH_2_O) 45.4 g Tris–HCl and adjust pH with HCl.
• *0.5 M Tris–HCl buffer, pH 6.8:* For 100 ml, dissolve (in ddH_2_O) 6 g Tris–HCl and adjust pH with HCl.
• *5% Separation electrophoresis gel:* For 8 ml, mix 4.5 ml ddH_2_O, 1.33 ml 30% acrylamide, 2 ml 1.5 M Tris pH 8.8, 80 μl 10% SDS, 80 μl 10% ammonium persulfate, and 8 μl TEMED.
• *3% Stacking electrophoresis gel:* For 5 ml, mix 3.17 ml ddH_2_O, 0.5 ml 30% acrylamide, 1.25 ml 0.5 M Tris pH 6.8, 50 μl 10% SDS, 50 μl 10% ammonium persulfate, and 5 μl TEMED.
• *CBB G-250 stain solution:* For 2 L, first dissolve 1 g CBB G-250 in 860 ml MetOH. Then add 140 ml of acetic acid and 1 L ddH_2_O.
• *Gel destain solution:* For 2 L, mix 200 ml MetOH and 140 ml acetic acid with 1.66 L ddH_2_O.
• *Electrophoresis sample buffer (4×):* For 9 ml, dissolve 0.02 g bromophenol blue and 0.8 g sodium dodecyl sulfate in 4 ml glycerol and 5 ml 0.5 M Tris–HCl buffer, pH 6.8.
• *Running buffer (10×):* For 1 L, dissolve (in ddH_2_O) 30.3 g Tris-base (final 0.25 M), 144.4 g glycine (final 1.92 M) and 10 g SDS (final 0.0347 M).
• *Running buffer (1×):* For 1 L, mix 100 ml of 10× running buffer with 900 ml ddH_2_O.
• *FOX (−Fe) reagent:* For 5 ml, dissolve (by stirring for 30 min) in ddH_2_O 0.0076 g XO, and add 0.07 ml sulfuric acid. Centrifuge for 5 min, at RT and at 13,000 *g* and collect the supernatant. The reagent can be stored at −20°C.
• *FOX (+Fe) reagent:* For 1 ml, dissolve (by vortexing) in 1 ml FOX (−Fe^2+^) reagent 0.0015 g ammonium ferrous sulfate. Prepare fresh.
• *20 mM SDS + 0.18% PEG-6000 + 0.1 M NaOH solution:* For 50 ml, dissolve (in ddH_2_O) 0.288 g SDS, 0.09 g PEG-6000 and 0.5 ml 10 M NaOH.
• *20 mM SDS + 0.18% PEG-6000:* For 50 ml, dissolve (in ddH_2_O) 0.288 g SDS and 0.09 g PEG-6000.
• *20 mM SDS:* For 50 ml, dissolve (in ddH_2_O) 0.288 g SDS.
• *Solution A:* For 0.75 ml, mix 625 μl TCA 100% with 125 μl 12 M HCl.
• *Solution B:* For 1 ml, dissolve (by vortexing) in 1 ml 0.2 M NaOH 0.025 g TBA.
• *TBA reagent:* For 1 ml, mix 0.5 ml solution A with 0.5 ml solution B.
• *TBA solvent:* For 1 ml, mix 0.5 ml solution A with 0.5 ml 0.2 M NaOH.
• *10 M NaOH:* For 100 ml, dissolve (in ddH_2_O) 40 g NaOH.
• *0.2 M NaOH:* For 10 ml, mix 9.8 ml ddH_2_O with 0.2 ml 10 M NaOH.
• *0.1 M BHA:* For 2 ml, dissolve in 1.966 ml EtOH, 0.036 g BHA.
• *TCA 100%:* For 5 ml, dissolve (in ddH_2_O) 5 g TCA.
• *3.3% PEG-6000:* For 10 ml, dissolve (in ddH_2_O) 0.33 g PEG-6000.
• *ApoB100 1 μg/μl:* For 0.5 ml, dissolve 500 μg pure apoB100 in 500 μl 20 mM SDS + 0.18% PEG-6000.

## 3. Methods

**FIGURE 2:**
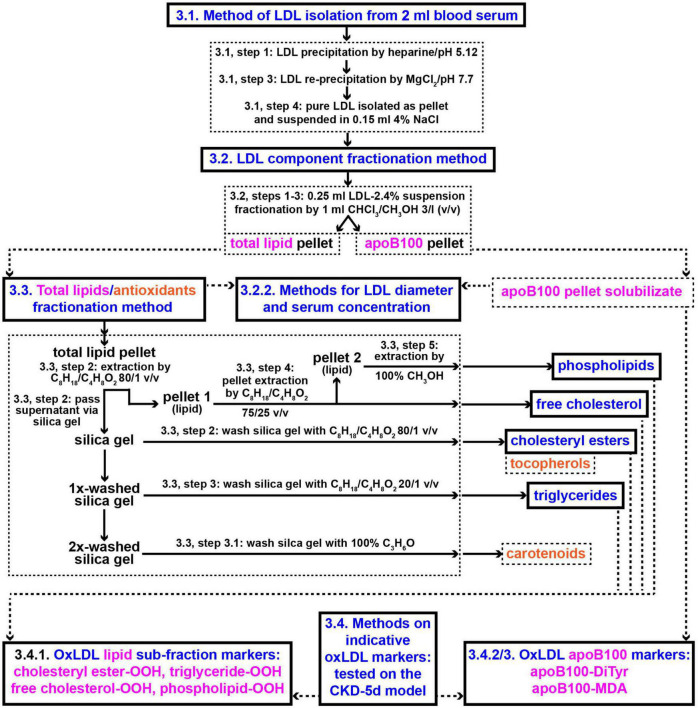
Methods outline protocols for the development of new clinical markers for CVD risk assessment based on oxLDL. Numbers in panels designate protocols’ sub-sections and steps in section “Methods”. CHCl_3_/CH_3_OH, chloroform/methanol; C_8_H_18_/C_4_H_8_O_2_, iso-octane/ethyl acetate; C_3_H_6_O, acetone.

The methods employed in the present study involve: (a) Protocols for isolation *from blood serum* of LDL particles (LDL-P) and their diameter size determination, (b) protocols for LDL fractionation into its main components: apoB100, cholesteryl esters, triglycerides, free cholesterol, phospholipids, carotenoids, and tocopherols, and (c) assays for the quantification of the following LDL components’ certain specific oxidative modifications: apoB100 malondialdehyde (apoB100-MDA) and dityrosines (apoB100-DiTyr), and the lipid hydroperoxides (-OOH) cholesteryl ester-OOH, triglyceride-OOH, free cholesterol-OOH, and phospholipid-OOH. These methods are diagrammatically outlined in Figure 2.

### 3.1. LDL isolation method (∼3 h for 12 samples)

Blood is separated into serum, is supplemented with 1/1 mM BHA/BHT (using the 400/400 mM BHA/BHT solution; to protect LDL from oxidation during isolation and handling), and can be either used immediately to isolate LDL, or stored frozen at −80°C. LDL is isolated from serum by combined modifications of the heparin-citrate precipitation at pH 5.12 (32), and the MgCl_2_-precipitation (33) methods. The innovation of this method is the isolation of LDL *via* two-step sequential precipitation: (1) by heparin, which removes most contaminating serum proteins (32); (2) by Mg^2+^, combined with an intermediate step of LDL solubilization in 4% NaCl which eliminates any remnant serum proteins (32, 33). These steps neutralize matrix effects mostly through ion suppression (34, 35). The protocol steps are as follows:

1. One milliliter serum sample is mixed (in a 15-ml glass Corex centrifuge tube) with 10 ml 64 mM citrate buffer, pH 5.12, to which are added 150 μl 5 KU/ml heparin stock (final 75 U/ml) and 25 μl 400/400 mM BHA/BHT solution (final 1/1 mM BHA/BHT), and incubated at 25°C for 10 min. The crude LDL precipitate is collected by centrifugation for 10 min at 10,000 *g* at 25°C (using a refrigerated centrifuge).
  *Notes:* (1) Higher centrifugation speeds can be used as long as 25°C is kept constant to ensure citrate buffer pH stability. (2) At pH 5.12, VLDL and HDL particles remain in solution, whereas LDL particles are selectively precipitated (32, 36).
2. The LDL precipitate in the tube (located at the bottom and lengthwise of the tube) is washed twice with 1 ml Hepes, pH 7.2, containing 1/1 mM BHA/BHT (by adding 2.5 μl 400/400 mM BHA/BHT), each time by scraping, with a metallic spatula, the inner wall of the tube toward its bottom, followed by centrifugation at 10,000 *g* for 5 min at 25°C. After each wash the supernatant is discarded.
3. Small quantities of serum proteins that may have contaminated the 2×-washed LDL precipitate are removed as follows: The 2×-washed LDL pellet is dissolved in 0.2 ml 4% NaCl, containing 1/1 mM BHA/BHT (by adding 4 μl 50/50 mM BHA/BHT), and to the resulting solution are sequentially added 10 ml 20 mM Tris, pH 7.7 (containing 1/1 mM BHA/BHT, by adding 25 μl 400/400 mM BHA/BHT), and 0.3 ml 5 M MgCl_2_ (final 143 mM), followed by incubation for 10 min in an ice-water bath, and precipitation of the pure LDL pellet by centrifugation at 10,000 *g* for 15 min at 4°C. It is important to withdraw (and discard) the supernatant immediately (by, e.g., a glass Pasteur pipette) to avoid resuspension of the LDL pellet.
  *Note:* MgCl_2_ concentration set at exactly 143 mM is crucial for LDL precipitation to take place; small increases (≥188 mM) result in ineffective precipitation, possibly related to a salting-out effect. That is, at MgCl_2_ concentrations ≥188 mM LDL particles are not precipitated (MgCl_2_ is not chelated with the phosphate groups of the LDL phospholipids), since Cl^–^ compete with LDL phospholipid PO_4_^–^ group, and “steal” the phospholipid-bound Mg^+2^, thus preventing LDL precipitation.
4. The resulting LDL pellet from *step 3* is washed with 1 ml Hepes, pH 7.2, containing 1/1 mM BHA/BHT (by adding 2.5 μl 400/400 mM BHA/BHT), and centrifuged at 10,000 *g* for 5 min at 4°C, followed by supernatant discarding. The resulting pellet is resuspended in 1 ml Hepes + 1/1 mM BHA/BHT (by mild suction using a 1 ml pipette tip), transferred in a 1.5-ml microcentrifuge tube, and centrifuged at 10,000 *g* for 5 min at 4°C, followed by supernatant discarding. At this point, the resulting pure LDL pellet can be either stored frozen at −80°C or suspended in 0.15 ml 4% NaCl (by vortexing) and subjected to the following method (in section 3.2).

### 3.2. Method for LDL fractionation into apoB100 and total lipids plus antioxidants (∼1.5 h for 12 samples)

Low-density lipoprotein particles in the NaCl-suspension are initially fractionated into their protein and total lipid + antioxidant components: the protein fraction consists of a single protein (apoB100), the total LDL lipid + antioxidant fraction consists of cholesteryl esters, triglycerides, free cholesterol, and phospholipids together with the antioxidant components carotenoids and tocopherols. Fractionation of the two aforementioned components is achieved by a modification of a previously developed lipid extraction method (37) as follows:

1. To the 0.15 ml purified LDL suspension (*step 4*, section 3.1), are added 0.1 ml 50 mM Pi buffer, pH 7.4, adjusted to contain 1/1 mM BHA/BHT (by adding 5 μl 50/50 mM BHA/BHT).
2. The resulting 0.25 ml LDL suspension is delipidated with 1 ml CHCl_3_/MetOH 3:l (v/v), containing 1/1 mM BHA/BHT (by adding 2.5 μl 400/400 mM BHA/BHT), by 1 min-vortexing, followed by centrifugation at 13,000 *g* for 5 min at 4°C, which forms an upper (aqueous) phase, a middle phase (containing the apoB100 protein disc) and a lower yellowish chloroform phase (total lipids, plus carotenoids, and tocopherols). The lower chloroform phase is collected (into a 2-ml microcentrifuge tube). To the remaining aqueous and apoB100 disc layer phases, 0.75 ml CHCl_3_ are added (containing 1/1 mM BHA/BHT), followed by 1 min-vortexing and centrifugation at 13,000 *g* for 5 min at 4°C. The upper aqueous phase is discarded and the lower CHCl_3_ phase is combined with the initial CHCl_3_ phase and vacuum-dried as dry extract of total LDL lipids, which are either stored frozen at −80°C, or immediately fractionated in their main classes (cholesteryl esters, triglycerides, free cholesterol, and phospholipids), and also the co-extracted carotenoids and tocopherols.
3. The chloroform-washed apoB100 pellet from *step 2* is washed 3× in 0.55 ml ice-cold acetone as follows: the apoB100 pellet is initially mixed with 50 μl ice-cold acetone and frittered into small pieces (with the narrow tip of a metallic spatula or with a glass rod, to facilitate its solubilization in a subsequent step). Then, another 0.5 ml of ice-cold acetone are added to the loose apoB100 acetone suspension, followed by 30-s mild vortexing and centrifugation at 13,000 *g* for 5 min at 4°C. After each wash the acetone supernatant is discarded and the 3×-acetone-washed apoB100 is vacuum-dried, collected in the (previously detached/pre-weighted) cap of a 1.5-ml microtube by inversion, and accurately weighted using a semi-micro analytical balance (0.1 mg sensitivity). At this point, the resulting apoB100 pellet can be stored at −80°C, or dissolved in ∼200 μl 20 mM SDS + 0.18% PEG-6000 (using a glass rod). For apoB100 solubilization, the protein pellet must be incubated for several minutes in 20 mM SDS + 0.18% PEG-6000 in order to be thoroughly impregnated. By doing so, the majority of the protein pellet is solubilized. Following centrifugation at 13,000 *g* for 1 min at 4°C, the soluble supernatant is collected, while a small insoluble, gel-like, remnant (possibly made up of serum protein impurities) is discarded.

#### 3.2.1. LDL purity assessment

The purity of the delipidated apoB100 (from *step 3*, section 3.2) is assessed *via* SDS-PAGE electrophoresis (5% polyacrylamide separating gel, 3% polyacrylamide 3 mm stacking gel, run for approximately 2 h at 120 V, 236 mA, 48 W). The gel is loaded by 30 μl LDL-isolated apoB100 and pure apoB100 (control), each added as duplicates of 10 and 20 μg, made by diluting their 1 μg/μl standard solutions with sample buffer containing 3% 2-mercaptoethanol. The 30 μl apoB100 loaded quantities are previously incubated for 3 min at 100°C and cleared by centrifugation at 13,000 *g*, at RT, for 4 min. The gel is stained using CBB G-250 stain solution, and apoB100 band purity is determined using Image Lab software by Bio-Rad.

#### 3.2.2. LDL particle size and serum concentration determination

The aim of this experiment is to provide evidence for the purity of LDL by determining its diameter (*d*), and comparing it against that reported in literature. LDL-P average diameter is indirectly calculated from the correspondence of 1 molecule apoB100 per one LDL-P, and its phospholipid circular surface (A), which is determined by its phospholipid numbers (determined by the phosphorus content of LDL total lipids and their 1:1 molar ratio), multiplied by the average phospholipid surface area 75 Å^2^ (38), and converted to *d*, presuming LDL-P forms a perfect sphere. Specifically, total lipids and apoB100 are isolated from section 3.2, *steps 2, 3*, and the dry weight of apoB100 is converted to apoB100 moles [by its MW 550 kDa (39, 40)], while the total number of phospholipids in the total lipid fraction is quantified by the phosphorus (P) content method (41), and their division gives the number of phospholipids per LDL-P. The same P content is determined for the phospholipid fraction isolated from total lipids in section 3.3, *step 5*. Number of phospholipids per LDL-P is, then, converted to its surface by multiplication with surface area (75 Å^2^) per phospholipid, which is used to calculate the *d* [by the formula A = π(*d*/2)^2^] corresponding to the circular LDL-P. LDL-P serum concentration (nmoles/L) is determined from apoB100% purity, weight and MW 550 kDa.

### 3.3. Method for sub-fractionation of LDL total lipids, carotenoids and tocopherols (∼4 h for 12 samples)

Total lipid fraction from *step 2*, section 3.2 is further sub-fractionated into cholesteryl esters, free cholesterol, triglycerides, phospholipids, as well as in carotenoids and tocopherols, by a modification of a previously reported method (37), using silica gel as follows:

1. Silica gel preparation: approximately 0.27 g silica gel are activated (in a 2-ml microcentrifuge tube) by 2×-wash with 1 ml 80:1 isooctane:EA as follows: the 1 ml 80:1 isooctane:EA/silica gel suspension is mildly mixed by hand inversion, followed by centrifugation at 13,000 *g* for 1 min at RT, with the supernatant discarded after each wash.
2. The LDL total lipid pellet (from *step 2*, section 3.2) is extracted 2× with 0.25 ml 80:1 v/v isooctane:EA, each time followed by vigorous vortexing and centrifugation at 15,000 *g* for 5 min at 4°C, resulting in a 2×-washed small clear pellet (seen by contrast difference), which contains the free cholesterol and phospholipid fractions. The combined (0.5 ml) yellowish supernatant is mixed with the wet 0.27 g activated silica gel from *step 1* and the tube is stirred by hand inversion, followed by centrifugation at 15,000 *g* for 5 min at 4°C, and the supernatant is collected. The silica gel pellet is washed 3× with 1 ml of 80:1 isooctane:EA as follows: 1 ml 80:1 isooctane:EA is added in the tube, stirred by hand inversion, and centrifuged at 15,000 *g* for 5 min at 4°C, the supernatant is collected, and the resulting silica gel pellet undergoes the same process two more times. The 3×-washed silica gel pellet (of yellowish color) is saved as it contains the LDL-triglyceride fraction (see *step 3*). Then, the combined supernatants (∼3.5 ml) are vacuum-dried, with the dry pellet being the LDL cholesteryl ester fraction containing also tocopherols [as being soluble in 80:1 isooctane:EA (42)]. Tocopherols can be measured by available assays (43–46), besides by the commercial kits. The LDL cholesteryl ester fraction can be stored at −80°C, or dissolved in 100 μl 80:1 isooctane:EA (by vortexing) to measure cholesteryl ester peroxidation (see section 3.4.1).
3. LDL triglycerides bound to the 3×-washed silica gel pellet (from *step 2*), are extracted by 3×-washing the pellet, each time with 1 ml 20:1 isooctane:EA by hand inversion, and centrifugation at 15,000 *g* for 5 min at 4°C. The combined supernatants (∼3 ml) are vacuum-dried, with the dry pellet being the LDL triglyceride fraction, which can be stored at −80°C, or dissolved in 100 μl 20:1 isooctane:EA (by vortexing) to measure triglyceride peroxidation (see section 3.4.1). The resulting silica gel pellet remains yellowish (as in *step 2*), which is due to bound carotenoids, as verified by their spectral identification (Figure 5) after extraction as in *step 3.1*.
  3.1 LDL carotenoids are extracted from silica by 2×-wash with 1 ml 100% acetone (by tube hand inversion), each time followed by centrifugation at 15,000 *g* for 5 min at 4°C. The combined supernatants (∼2 ml) are vacuum-dried, and the resulting dry yellowish pellet can be stored at −80°C, or dissolved in 0.3 ml hexane (by vortexing) for spectral analysis. Carotenoids are quantified by the equation: Total carotenoids (in μM) = 21.359 × Absorbance_448 nm_ − 0.1053, where Absorbance_448 nm_ = Absorbance_448 nm_ − Absorbance_550 nm_ (47).
4. The small clear pellet from *step 2* is extracted, for free cholesterol, with 2 × 0.25 ml 75:25 isooctane:EA, followed by vortexing and centrifugation at 15,000 *g* for 5 min at 4°C, resulting in a 2×-washed small clear pellet (seen by contrast difference), which contains the LDL phospholipid fraction. The combined supernatants (∼0.5 ml) are vacuum-dried, with the dry pellet being the LDL free cholesterol fraction, which can be stored at −80°C, or dissolved in 100 μl 75:25 isooctane:EA (by vortexing) to measure cholesterol peroxidation (see section 3.4.1).
5. The small clear pellet from *step 4* is extracted, for phospholipids, with 2 × 0.25 ml MetOH, followed by vortexing and centrifugation at 15,000 *g* for 5 min at 4°C, ending up with no pellet remnant formation. The combined supernatants (∼0.5 ml) are vacuum-dried, with the dry pellet being the LDL phospholipid fraction, which can be stored at −80°C, or dissolved in 100 μl MetOH (by vortexing) to measure phospholipid peroxidation (see section 3.4.1).

**FIGURE 3:**
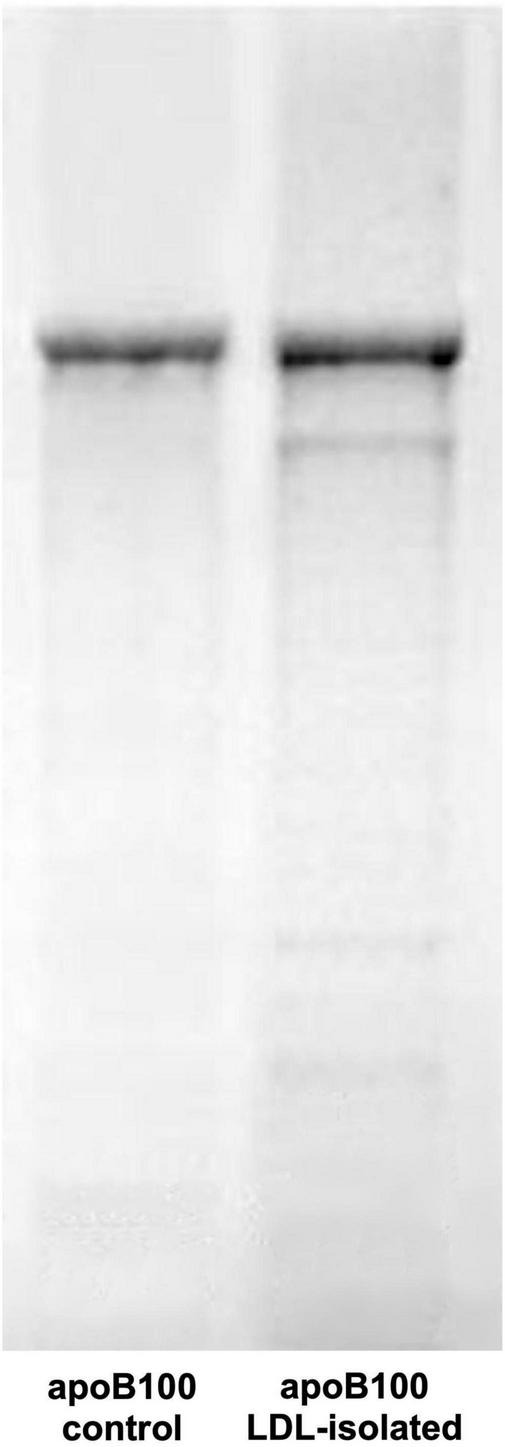
SDS-PAGE electrophoresis of human LDL apoB100. The gel shows apoB100 isolated from LDL (sample band in **right** gel column) against pure apoB100 (control band in **left** gel column).

**FIGURE 4:**
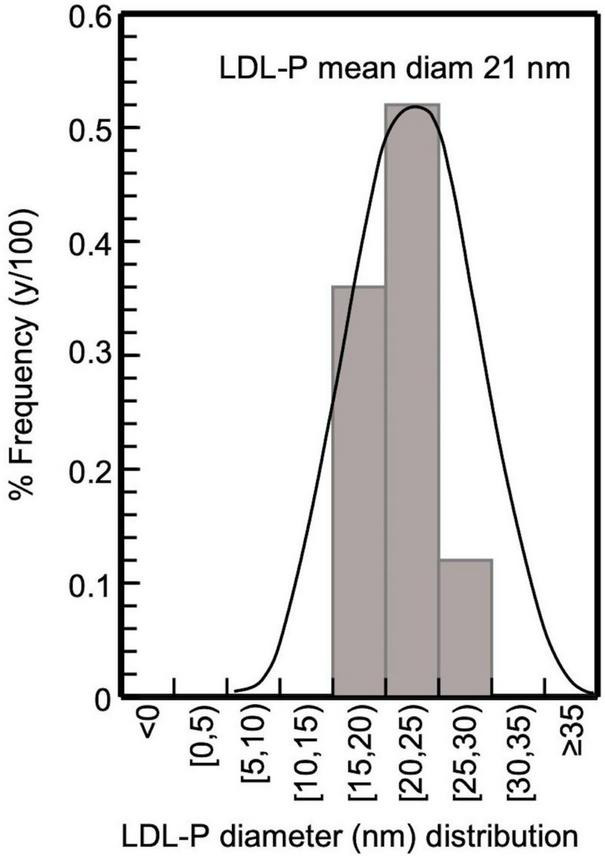
Frequency histogram of LDL-P diameter in CKD-5d patients participating in the present study; 32 subjects with LDL-P 20–25 nm, 22 with 15–20 nm, and 7 with 25–30 nm, giving an average 21 nm.

**FIGURE 5:**
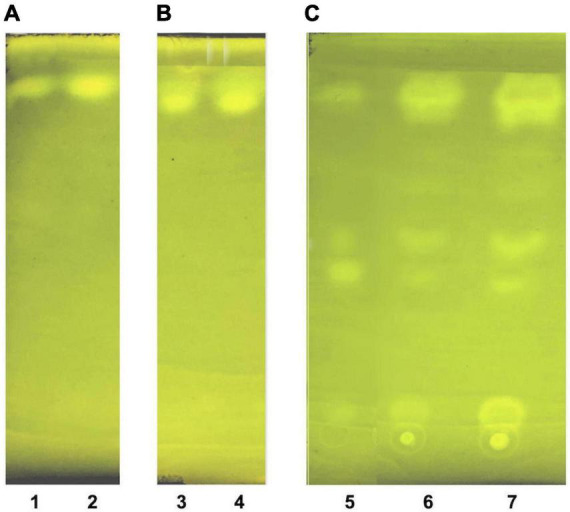
Thin layer chromatography of LDL lipid sub-fractions. **(A)** LDL cholesteryl esters (lane 1: 1× moles, lane 2: 2× moles); **(B)** LDL triglycerides (lane 3: 1× moles, lane 4: 2× moles); and **(C)** LDL free cholesterol (lane 5: pure cholesterol standard solution, lane 6: 1× moles LDL free cholesterol, lane 7: 2× moles LDL free cholesterol).

#### 3.3.1. LDL lipid sub-fraction purity assessment by thin layer chromatography and phosphorus quantification

Lipid sub-fractions isolated in section 3.3 are identified and tested for purity by an extended modification of a previously reported thin layer chromatography (TLC)-based method (48), as follows: Before sample loading, TLC aluminum silica gel 60 sheets (e.g., 5 × 5 cm) are previously dried at 60°C in an oven for 1 h, then washed out (for impurities) by immersion in the developing solvent (hexane:diethyl ether:acetic acid 50:50:1 v/v/v) until fully exposure, and finally air-dried at 60°C. Lipid sub-fraction samples (∼2 μl) are loaded on the pretreated TLC plates, and exposed to the developing solvent, followed by air-drying at 60°C, and then immersed in a beaker containing 0.005% Rhodamine 6G solution for sample band staining, plate air-drying and band visualization by an ultra-violet lamp at 254 nm. Furthermore, the identity of the isolated LDL phospholipid sub-fraction -and the absence of phospholipids from the previously mentioned three LDL lipid sub-fractions is verified by phosphorus quantification in each sub-fraction, using a previously reported method (41).

### 3.4. Methods for measuring selected specific oxidative modifications on oxLDL components

Peroxidized lipids are selected as the mostly OS-susceptible general markers [due to their self-propagated generation mechanism (49)] to assess on oxLDL lipid component sub-fractions the initially formed hydroperoxides (LOOH) (50). LOOH are subsequently oxidatively decomposed to aldehyde products, with more pronounced the very reactive malondialdehyde (MDA), which attacks proteins (Pr) forming PrMDA products (50). In that respect, we assess oxidation of the apoB100 LDL component by the selection of apoB100-MDA as a valid marker of apoB100 oxidation, forming apoB100-MDA bound. Similarly, DiTyr are also selected as a representative apoB100 oxidation marker (apoB100-DiTyr) because it is associated with the molecular pathology of dityrosine cross-links in proteins (51).

#### 3.4.1. Hydroperoxide determination in the isolated LDL lipid sub-fractions

Measurement of LOOH in the solubilized LDL lipid sub-fractions of cholesteryl esters (cholesteryl ester-OOH), triglycerides (triglyceride-OOH), free cholesterol (free cholesterol-OOH), and phospholipids (phospholipid-OOH), sub-fractionated from LDL isolated from 2 ml blood serum (following sections 3.1 to 3.3), is performed by minor modification of a previously reported assay, developed by our group (50). For each of the four lipid sub-fractions, are prepared different dilutions (μl) of sample (S_xμl_), and of control sample blank (SB_xμl_) and reagent blank (RB_xμl_), composed of mixtures of reagents (placed in 1.5-ml Eppendorf tubes) as shown in Tables 1–4. The net absorbance (A) of each sample is calculated using the following formula: Net A = (S_A_ − SB_A_) − [RB (+Fe^2+^)_A_ − RB (−Fe^2+^)_A_]. Finally, net A is converted to cumene hydroperoxide (cum-OOH) nmole equivalents using a cum-OOH standard curve, using solvents same as those used in the assay, and at their maximum volumes. Lipid sub-fraction hydroperoxide cum-OOH equivalents are expressed as cum-OOH nmole/mg apoB100 (also isolated from 2 ml blood serum).

**TABLE 1:** Assay set up for LOOH determination in LDL cholesteryl esters.

Reaction reagents (numbers show μl)	S_15 μl_	S_30 μl_	SB_15 μl_	SB_30 μl_	RB_15 μl_ (+Fe^2+^)	RB_30 μl_ (+Fe^2+^)	RB_30 μl_ (−Fe^2+^)
Cholesteryl esters	15	30	15	30	–	–	–
80:1 isooctane:EA	–	–	–	–	15	30	30
MetOH	235	220	235	220	235	220	220
FOX (**+**Fe^2+^)	50	50	–	–	50	50	–
FOX (−Fe^2+^)	–	–	50	50	–	–	50

Incubation for 30 min at RT, followed by centrifugation at 13,000 *g* for 5 min at 4°C, and absorbance (A) recording at 560 nm. The optical absorption of RB (−Fe^2+^) is not affected by 80:1 isooctane:EA, meaning RB with the highest 80:1 isooctane:EA volume [RB_30_ (−Fe^2+^)] is tested. Each RB_xμl_ is prepared in triplicate. If sample size is restrictive, control SB can be omitted since its A value matches that of RB (−Fe^2+^).

**TABLE 2:** Assay set up for LOOH determination in LDL triglycerides.

Reaction reagents (numbers show μl)	S_10 μl_	S_20 μl_	SB_10 μl_	SB_20 μl_	RB_10 μl_ (+Fe^2+^)	RB_20 μl_ (+Fe^2+^)	RB_20 μl_ (−Fe^2+^)
Triglycerides	10	20	10	20	–	–	–
20:1 isooctane:EA	–	–	–	–	10	20	20
MetOH	240	230	240	230	240	230	230
FOX (+Fe^2+^)	50	50	–	–	50	50	–
FOX (−Fe^2+^)	–	–	50	50	–	–	50

Incubation for 30 min at RT, followed by centrifugation at 13,000 *g* for 5 min at 4°C, and absorbance (A) recording at 560 nm. The optical absorption of RB (−Fe^2+^) is not affected by 20:1 isooctane:EA, meaning RB with the highest 20:1 isooctane:EA volume [RB_20_ (−Fe^2+^)] is tested. Each RB_xμl_ is prepared in triplicate. If sample size is restrictive, control SB can be omitted since its A value matches that of RB (−Fe^2+^).

**TABLE 3:** Assay set up for LOOH determination in LDL free cholesterol.

Reaction reagents (numbers show μl)	S_20 μl_	S_40 μl_	SB_20 μl_	SB_40 μl_	RB_20 μl_ (+Fe^2+^)	RB_40 μl_ (+Fe^2+^)	RB_40 μl_ (−Fe^2+^)
Free cholesterol	20	40	20	40	–	–	–
75:25 isooctane:EA	–	–	–	–	20	40	40
MetOH	230	210	230	210	230	210	210
FOX (+Fe^2+^)	50	50	–	–	50	50	–
FOX (−Fe^2+^)	–	–	50	50	–	–	50

Incubation for 30 min at RT, followed by centrifugation at 13,000 *g* for 5 min at 4°C, and absorbance (A) recording at 560 nm. The optical absorption of RB (−Fe^2+^) is not affected by 75:25 isooctane:EA, meaning RB with the highest 75:25 isooctane:EA volume [RB_40_ (−Fe^2+^)] is tested. Each RB_xμl_ is prepared in triplicate. If sample size is restrictive, control SB can be omitted since its A value matches that of RB (−Fe^2+^).

**TABLE 4:** Assay set up for LOOH determination in LDL phospholipids.

Reaction reagents (numbers show μl)	S_100 μl_	RB (+Fe^2+^)	RB (−Fe^2+^)
Phospholipids	100*	–	–
MetOH	150	250	250
FOX (+Fe^2+^)	50	50	–
FOX (−Fe^2+^)	–	–	50

Incubation for 30 min at RT, followed by centrifugation at 13,000 *g* for 5 min at 4°C, and absorbance (A) recording at 560 nm. Each RB_xμl_ is prepared in triplicate. *All phospholipid solubilizate volume is used for increased sensitivity, thereby not using SB, given SB’s A value has been tested to be the same of that of RB (−Fe^2+^).

#### 3.4.2. MDA determination in apoB100

Measurement of MDA bound to apoB100 (apoB100-MDA) in the LDL apoB100 fraction (from LDL isolated from 2 ml blood serum, and solubilized as in *step 3* of section 3.2) is performed by modification of a previously reported assay, developed by our group (50). Before testing, ∼150 μl from the initial 200 μl apoB100 solubilizate are mixed with 1.5 μl 10 M NaOH (final 0.1 M NaOH), and incubated for 30 min at 60°C (in a water bath), so as to hydrolyze MDA from apoB100. For each NaOH-treated apoB100 solubilizate, are prepared different dilutions (in 20 mM SDS + 0.18% PEG-6000 + 0.1 M NaOH) of sample (S) and sample blank (SB), adjusted to 250 μl, and a reagent blank (RB), and are mixed (in 1.5-ml Eppendorf tubes) with certain assay reagents as shown in Table 5. Mixtures in Table 5 are incubated for 20 min at 100°C (in a water bath, while keeping the lids of the tubes open). Then, the formed reaction product is extracted in 0.3 ml ButOH by vortexing, which forms an upper phase upon centrifugation at 13,000 *g* for 5 min at RT. Subsequently, the upper ButOH phase is isolated and its fluorescent units (FU) are measured at ex/em 535/550 nm (setting at low sensitivity the spectrofluorometer in use). Net FU for each sample is calculated from the formula net FU = S_FU_ − SB_FU_ − RB_FU_, which is converted to MDA pmoles, using a corresponding standard curve of pure MDA (in concentrations ranging from 0.08 μM to 0.66 μM in 20 mM SDS + 0.18% PEG-6000 + 0.1 M NaOH), and expressed as MDA pmoles/mg apoB100 (also isolated from 2 ml blood serum).

**TABLE 5:** Assay set up for apoB100-MDA determination.

Reagents (μl)	S	SB	RB
ApoB100	250	250	–
20 mM SDS **+** 0.18% PEG-6000 **+** 0.1 M NaOH	–	–	250
0.1 M BHA	3	3	3
TBA reagent	50	–	50
TBA solvent	–	50	–

RB is prepared in triplicate.

#### 3.4.3. DiTyr determination in apoB100

For apoB100-DiTyr determination, 50–100 μg apoB100 are drawn from its solubilizate (*step 3* of section 3.2) are further diluted in 20 mM SDS to final 0.3 ml, and their FU are measured at ex/em 320/405 nm (setting at low sensitivity the spectrofluorometer in use) against a triplicate RB [containing 0.3 μl 20 mM SDS and 2 μl 3.3% PEG-6000 (final 0.022%; the maximum least fluorescence interfering PEG-6000 concentration)]. Sample DiTyr FU are converted to pmoles (per mg apoB100; isolated from 2 ml blood serum) *via* a standard curve of DiTyr [synthesized as previously reported in (52)], ranging from 0.56 to 6.8 μM, in final 0.3 ml, made in 20 mM SDS + 0.022% PEG-6000, and run against an RB containing 0.3 μl 20 mM SDS + 0.022% PEG-6000.

### 3.5. Application to CKD-5d patients versus controls

For the application of the aforementioned methodologies, 61 CKD-5d patients (20–94 years old) and 40 healthy, sex and age matched, control adults (23–67 years old) are recruited from the Department of Nephrology and Kidney Transplantation of the General University Hospital of Patras, Greece. Specific demographic data and the medical status for all patients are presented in Supplementary Table 1. Additionally, blood serum LDL-C is measured by a direct method (*via* LDL-cholesterol kit) in all subjects for comparison with the LDL-C calculated indirectly by the Friedewald equation (data not shown). Moreover, we test whether LDL-P size and serum concentration can be used to assess high risk for CVDs development in CKD-5d patients, using a receiver operator characteristic (ROC) curve (see also section 3.6). Particularly, we test if lower or higher LDL-P size and serum concentration values correlate with higher risk of CVDs development, the latter as assessed by LDL-C (measured by the LDL-cholesterol kit). For this purpose, we divide CKD-5d group into high risk (LDL-C >100 mg/dl) and low risk (LDL-C <100 mg/dl), according to existing guidelines for the LDL-C clinical marker in CVDs development risk assessment (12). For each patient LDL-P size and serum concentration are determined and ROC curves are generated.

From each patient, blood samples are drawn right before and after hemodialysis procedure, to test possible hemodialysis oxidation effects on LDL, while blood from control subjects is collected after overnight fasting. Participation in the study is voluntary. All subjects gave a written consent, after having been thoroughly briefed on the purposes of the present study. The protocol of the study is approved by the Scientific Committee of the General University Hospital of Patras (No. 353/02/09/2015). The employed experimental procedures are in accordance with the ethical standards of the Helsinki Declaration of 1975, as revised in 2013.

### 3.6. Statistical analysis

Data are presented as mean (*M*) ± standard deviation (SD), using IBM SPSS Statistics 26. A normality test (*Kolmogorov–Smirnov*) is performed on all numerical data. Regarding the comparison of oxLDL markers before and after hemodialysis (dependent samples), *the Wilcoxon signed-rank* test is performed for the not normally distributed data (for cholesteryl ester-OOH, triglyceride-OOH, free cholesterol-OOH, phospholipid-OOH, and apoB100-DiTyr), whereas the *paired samples* t*-test* is used for the normally distributed data (for apoB100-MDA). Regarding the comparison of oxLDL markers between control group and CKD-5d patient group (independent samples) that are not normally distributed, the *Mann–Whitney U* test is performed and the homogeneity of variance is checked and confirmed by the *Levene’s* test. In order to check for possible association between the medical conditions and the medication received by CKD-5d patients with oxLDL markers, the *Mann-Whitney U* test is also performed (since the variables are not normally distributed), and the homogeneity of variance is checked and confirmed by the *Levene’s* test. Furthermore, in order to examine possible statistically significant association between the tested oxLDL markers and CKD-5d patients’ age, years of hemodialysis, and the clinical biochemical markers (LDL-C, HDL-C, total cholesterol, triglycerides, CRP, vitamin D, iPTH, Ca, P, and CaxP), the *Linear Regression Analysis* is performed. Additionally, the *ROC analysis* is performed in order to assess whether LDL-P size and serum concentration could predict CVDs risk, as assessed by LDL-C in CKD-5d patients. The significance level for all tests is set at *p* ≤ 0.05.

Finally, the LOOH, apoB100-MDA and apoB100-DiTyr assays are analyzed statistically for precision, both with time (between-run, or between day repeatability) and during a single analytical run (within-run, within-day precision, or repeatability). LOOH, apoB100-MDA and apoB100-DiTyr value minimum statistical variations are determined by analyzing at least three successive dilutions of LDL sub-fractions and calculating their mean value. The variance of intermediate precision (σ^2^ total) is defined as the sum of between day variance (σ^2^ between)—associated with the day-to-day variation—and the variance of repeatability (σ^2^ within), and the within-day% coefficient variation is calculated as SD 100/mean.

## 4. Results

The present study uses innovative methods to readily isolate LDL, assess its particle size and fractionate its main components (apoB100, phospholipids, triglycerides, free cholesterol, and cholesteryl esters) to identify/quantify in them certain oxidative modifications as potential clinical markers of oxLDL status in atherosclerosis associated CVDs, with indicative application to CKD-5d patients.

### 4.1. Assessing LDL, apoB100, and lipid purity, and LDL-P size and serum concentration

The present study selectively isolates LDL from blood serum by a simple precipitation protocol, which combines heparin-citrate-based LDL precipitation at pH 5.12 (32) and MgCl_2_-based LDL precipitation at pH 7.7 (33). This protocol is of time-short duration (∼3 h for 12 samples), and isolates LDL with a recovery >90%, as already established for this kind of precipitation approach (32, 36), with a purity up to ∼90% (as assessed by SDS-PAGE electrophoresis; Figure 3), which also reflects that of LDL (since apoB100/LDL ratio is 1). LDL purity is also verified, indirectly, by the determination of the mean diameter of the LDL-P, performed for the first time by measuring the number of LDL phospholipids (*via* their phosphorus content), and subsequently determining LDL particle sphere area, by the mean surface of each phospholipid. This approach produces an average of ∼21 nm LDL-P diameter in CKD-5d patients (Figure 4), and is an additional indirect confirmation of our LDL’s purity since it falls within the 22–29 nm reported range (53). Moreover, the present study measures LDL-P serum concentration by a simple method based on apoB100 weight, and converted to nmoles by its MW (550 kDa) after correction for purity (90%). This gives an average of 1,330 ± 500 nmoles/L LDL-P serum concentration in CKD-5d patients, which falls within the range of the values determined by NMR (54, 55).

**FIGURE 6:**
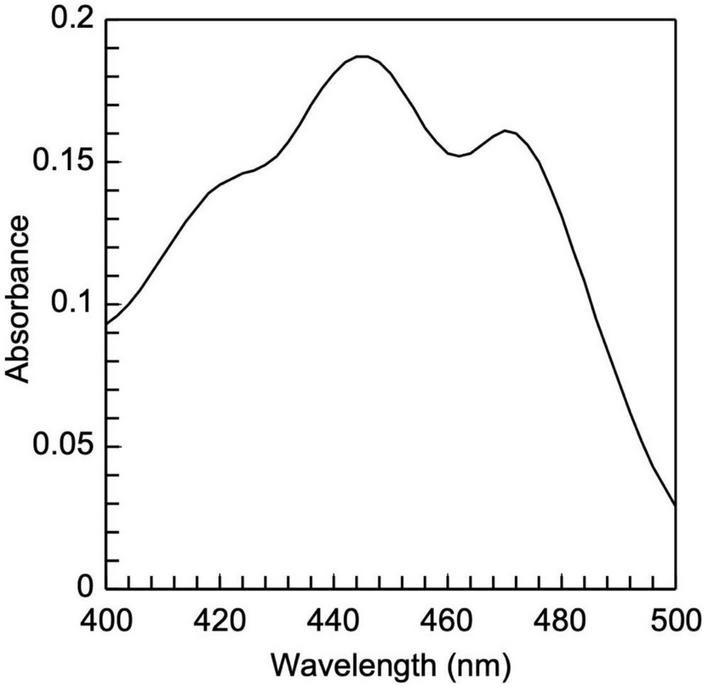
Low-density lipoprotein carotenoid absorbance spectrum. It is composed of two peaks, 448 and 470 nm, which are typical of carotenoids.

The present study also introduces innovative methodologies for the fractionation and purification of LDL’s main lipid sub-fractions: cholesteryl esters, triglycerides, free cholesterol, and phospholipids. The identity/purity of the former three is determined by TLC band mobility patterns (see section 3.3.1, Figure 5 and Supplementary Figure 1), while the purity of LDL phospholipids is confirmed *via* their single phosphorus content quantification (see section 3.3.1). Specifically, LDL cholesteryl esters (Figure 5A) and LDL triglycerides (Figure 5B) are 100% pure, as they migrate in single bands against background, whereas their identity is confirmed *via* the relative mobility (Rf) of their TLC bands in comparison with those previously reported (48, 56) (see Supplementary Figure 1). It should be noted that free cholesterol runs in four main TLC bands with minor ones in between (Figure 5C, lanes 6, 7), with same band number appearing even in pure cholesterol (Figure 5C, lane 5). This could be due to the different oxidized cholesterol forms (56), observed even in commercial cholesterol; up to 32 different due to its autoxidation upon storage (56). An additional, although indirect, criterion for the purity of the fractionated cholesteryl esters, triglycerides and free cholesterol is the absence of phosphorus upon testing (see section 3.3.1) (data not shown). Furthermore, LDL carotenoids (isolated in section 3.3, *step 3.1*) are indirectly verified by their absorbance spectrum (Figure 6), which is identical to a typical carotenoid absorbance spectrum reported in literature (47). Moreover, the isolation of carotenoids and tocopherols in separate fractions by the present study, facilitate the identification of their individual species (57, 58).

Having successfully sub-fractionated and identified apoB100 and the lipid sub-fractions of the isolated LDL, indicatively, from patients with CKD-5d, we evaluate on them (before and after their hemodialysis and versus controls) the levels of six indicative oxidative modifications (among others, Figure 1) as characteristic of oxLDL status.

### 4.2. Measurement of indicative specific oxidative modifications in LDL’s main components in CKD-5d patients before and after hemodialysis versus control subjects

The developed protocols are applied for the isolation and oxidative status characterization of the LDL of CKD-5d patients (versus controls subjects). The sub-fractionated LDL’s main components from CKD-5d patients before and after hemodialysis, are analyzed for the following six oxLDL-associated specific markers: cholesteryl ester-OOH, triglyceride-OOH, free cholesterol-OOH, phospholipid-OOH, apoB100-MDA, and apoB100-DiTyr. A slight increase in the levels of cholesteryl ester-OOH, triglyceride-OOH, phospholipid-OOH and apoB100-MDA, together with a slight decrease in the levels of free cholesterol-OOH, and apoB100-DiTyr are observed after hemodialysis. However, these slight changes are not statistically significant in both cases (see Supplementary Table 2). Specifically, the *Wilcoxon signed ranked* test reveals that: (a) cholesteryl ester-OOH levels do not differ significantly before and after hemodialysis, *Z* = −1.20, *p* = 0.23; (b) triglyceride-OOH levels do not differ significantly before and after hemodialysis, *Z* = −1.70, *p* = 0.87; (c) free cholesterol-OOH levels do not differ significantly before and after hemodialysis, *Z* = −0.19, *p* = 0.85; (d) phospholipid-OOH levels do not differ significantly before and after hemodialysis, *Z* = −0.09, *p* = 0.93; (e) apoB100-DiTyr levels do not differ significantly before and after hemodialysis, *Z* = −1.84, *p* = 0.07. Finally, the *paired sample* t*-test*, conducted to compare the levels of apoB100-MDA before and after hemodialysis, shows no significant difference in apoB100-MDA levels before and after hemodialysis, *t*(60) = −0.7, *p* = 0.49.

In light of the above results, we focus our investigation in comparing the aforementioned markers for CKD-5d patients before hemodialysis (CKD5d-BH) with the control group. A statistically significant increase is observed in the levels of triglyceride-OOH and free cholesterol-OOH (1.5- and 2.5-fold, respectively) in the CKD-5d patients versus the control group, while a statistically insignificant slight increase is observed in cholesteryl ester-OOH, phospholipid-OOH, apoB100-MDA and apoB100-DiTyr (Table 6). Specifically, the *Mann–Whitney U* test reveals that: (a) cholesteryl esters-OOH levels do not differ significantly between CKD-5d group and the control group, *U* = 599.00, *Z* = −0.12, *p* = 0.90; (b) triglyceride-OOH levels are significantly higher in CKD-5d group compared to the control group, *U* = 391.00, *Z* = −2.40, *p* = 0.02; (c) free cholesterol-OOH levels are significantly higher in CKD-5d group compared to the control group, *U* = 293.00, *Z* = −3.47, *p* = 0.01; (d) phospholipid-OOH levels do not differ significantly between CKD-5d group and the control group, *U* = 542.50, *Z* = −0.74, *p* = 0.46; (e) apoB100-MDA levels do not differ significantly between CKD-5d group and the control group, *U* = 328.50, *Z* = −2.21, *p* = 0.1; and (f) apoB100-DiTyr levels do not differ significantly between CKD-5d group and the control group, *U* = 471.50, *Z* = −1.52, *p* = 0.13.

**TABLE 6:** Oxidation markers of LDL lipid sub-fractions and apoB100 in CKD-5d patients before hemodialysis versus the control group.

Oxidative marker	Subjects	Value	*P*-Value
Cholesteryl ester-OOH	CKD5d-BH	0.95 (±0.76)	0.90
	Control	0.83 (±0.47)	
Triglyceride-OOH	CKD5d-BH	1.52 (±1.04)	0.02
	Control	1.03 (±0.74)	
Free cholesterol-OOH	CKD5d-BH	0.40 (±0.10)	0.01
	Control	0.16 (±0.06)	
Phospholipid-OOH	CKD5d-BH	0.18 (±0.19)	0.46
	Control	0.12 (±0.06)	
apoB100-MDA	CKD5d-BH	15.68 (±5.96)	0.1
	Control	14.40 (±5.68)	
apoB100-DiTyr	CKD5d-BH	10.85 (±5.40)	0.13
	Control	8.79 (±2.78)	

Values are presented as mean (*M*) and standard deviation (SD). Before hemodialysis serum testing is designated CKD5d-BH. LDL sub-fraction-OOH marker is expressed as cum-OOH nmole equivalents/mg apoB100, apoB100-MDA marker as MDA pmole/mg apoB100, and apoB100-DiTyr marker as DiTyr pmole/mg apoB100.

The increased levels of triglyceride-OOH and free cholesterol-OOH markers (Table 6) are statistically investigated for correlation with patients’ underlying medical status. Specifically, the *Mann–Whitney U* test is performed to investigate whether any of the CKD-5d patients’ underlying medical conditions and sex, correlate with the increase in the levels of these oxLDL markers. Such correlation does not exist according to such statistical analysis (statistical details are shown in Supplementary Table 3). Similarly, the *Mann–Whitney U* test is performed to determine any possible correlation of the medication received by CKD-5d patients with oxLDL triglyceride-OOH and free cholesterol-OOH marker levels. It is found that the medicines taken by CKD-5d patients do not correlate with these elevated oxLDL markers (statistical details are shown in Supplementary Table 4). Focusing on statin medication, it is worth noting that no statistical correlation is observed in all six tested oxLDL markers between CKD-5d patients under statin treatment (CKD-5dSt; *N* = 22) and under no statin treatment (CKD-5dnoSt; *N* = 39) (Table 7).

**TABLE 7:** Oxidation markers of LDL free cholesterol and triglyceride sub-fractions and apoB100 in CKD-5d patients under ± statin treatment.

O xidative marker	Patient group	Value	*P*-Value
Cholesteryl ester-OOH	CKD-5dSt	0.84 (±0.56)	0.58
	CKD-5dnoSt	0.73 (±0.86)	
Triglyceride-OOH	CKD-5dSt	1.34 (±0.91)	0.34
	CKD-5dnoSt	1.62 (±1.11)	
Free cholesterol-OOH	CKD-5dSt	0.34 (±0.25)	0.54
	CKD-5dnoSt	0.43 (±0.39)	
Phospholipid-OOH	CKD-5dSt	0.14 (±0.15)	0.18
	CKD-5dnoSt	0.19 (±0.21)	
apoB100-MDA	CKD-5dSt	17.00 (±5.99)	0.1
	CKD-5dnoSt	15.00 (±5.92)	
apoB100-DiTyr	CKD-5dSt	8.98 (±3.93)	0.15
	CKD-5dnoSt	9.11 (±3.87)	

CKD-5d patients under statin/no statin treatment (CKD-5dSt/CKD-5dnoSt). Values are presented as mean (*M*) and standard deviation (SD). LDL sub-fraction-OOH marker is expressed as cum-OOH nmole equivalents/mg apoB100, apoB100-MDA marker as MDA pmole/mg apoB100, and apoB100-DiTyr marker as DiTyr pmole/mg apoB100.

We further investigate whether years of hemodialysis and age of CKD-5d patients correlate with the increase in the levels of oxLDL triglyceride-OOH and free cholesterol-OOH markers, using *Simple Linear Regression* analysis. The statistical analysis shows that years of hemodialysis do not correlate with triglyceride-OOH levels (β = 0.05, *t* = 1.85, *p* = 0.07), since years of hemodialysis explain 4% of the variance [*R*^2^ = 0.04, *F*(1,59) = 3.42, *p* = 0.07]. Similarly, there is no statistically significant correlation of years of hemodialysis with the levels of free cholesterol-OOH (β = 0.006, *t* = 0.60, *p* = 0.55), since years of hemodialysis explain only 1% of the variance [*R*^2^ = 0.01, *F*(1,59) = 0.36, *p* = 0.55]. Same statistical analysis shows that age does not correlate with triglyceride-OOH levels (β = 0.03, *t* = 0.37, *p* = 0.71), since age explains only 2% of the variance [*R*^2^ = 0.02, *F*(1,59) = 0.14, *p* = 0.71]. Similarly, there is no statistically significant correlation of age with the levels of free cholesterol-OOH (β = 0.003, *t* = 0.01, *p* = 0.99), since age explains only 2% of the variance [*R*^2^ = 0.02, *F*(1,59) = 0.00, *p* = 0.99].

**FIGURE 7:**
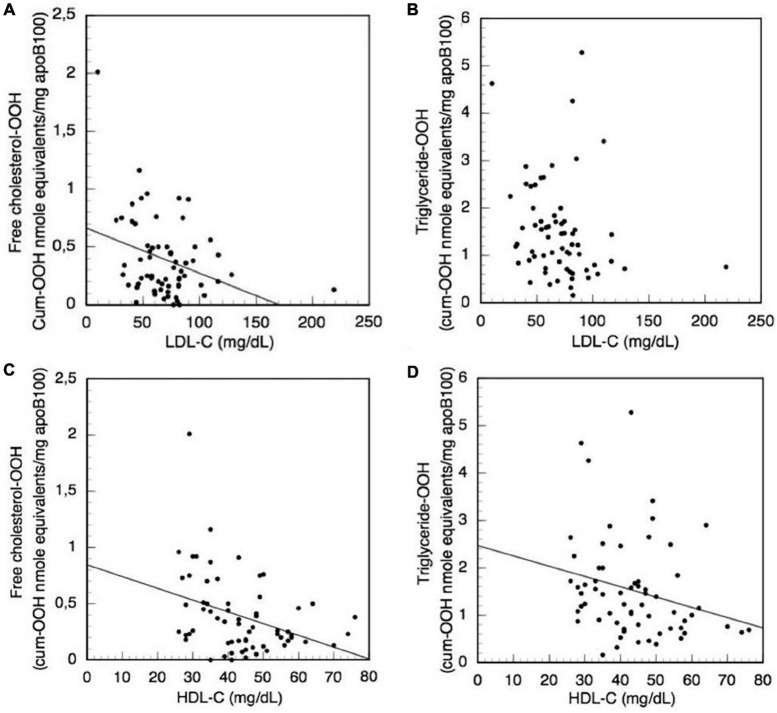
Correlation of the clinical biochemical markers LDL-C and HDL-C with LDL triglyceride-OOH and free cholesterol-OOH levels in CKD-5d patients. **(A)** LDL-C with free cholesterol-OOH; **(B)** LDL-C with triglyceride-OOH; **(C)** HDL-C with free cholesterol-OOH; and **(D)** HDL-C with triglyceride-OOH.

Moreover, the levels of the biochemical clinical markers LDL-C, total cholesterol, HDL-C, triglycerides, vitamin D, CRP, iPTH, Ca, P, and CaxP are correlated with the levels of oxLDL triglyceride-OOH and free cholesterol-OOH markers in CKD-5d patient group, using also *Simple Linear Regression* analysis. It is found that total cholesterol, triglycerides, vitamin D, CRP, iPTH, Ca, P, and CaxP do not significantly correlate with the levels of the aforementioned oxLDL markers. Focusing on the LDL-C clinical marker, since its increased levels are used to assess high risk for CVDs, we investigate (using *Simple Linear Regression* analysis) its possible association with the increased triglyceride-OOH and free cholesterol-OOH levels in CKD-5d patients (Figures 7A, B). The plotted data show that LDL-C levels do not significantly correlate with triglyceride-OOH levels (β = −0.21, *t* = −1.72, *p* = 0.91), since LDL-C levels explain only 3% of the variance [*R*^2^ = 0.03, *F*(1,62) = 2.94, *p* = 0.09] (Figure 7B). In contrast, there is a statistically significant inverse correlation of LDL-C with the levels of free cholesterol-OOH (β = −0.004, *t* = −2.83, *p* = 0.006), since LDL-C levels explain 10% of the variance [*R*^2^ = 0.10, *F*(1,62) = 8.00, *p* = 0.006] (Figure 7A). Looking in more detail at this inverse LDL-C versus free cholesterol-OOH correlation, it can be observed that some CKD-5d patients with low LDL-C levels (<60 mg/dl) have high levels of free cholesterol-OOH, which are higher than those of some CKD-5d patients with high LDL-C levels (> 100 mg/dl). It should be also noted our finding that LDL-C measured by the Friedewald equation (Figures 7A, B) is actually overestimated 1.42 times (on average, with range 1.01–3.55), when compared to the values obtained by the LDL-C specific method.

Focusing on the HDL-C clinical marker, we investigate its possible association with the increased triglyceride-OOH and free cholesterol-OOH levels in CKD-5d patients (Figures 7C, D), because the increased levels of HDL-C are used as indicator for low CVDs risk. The plotted data are subjected to *Simple Linear Regression*, and show that HDL-C levels present a statistically significant inverse correlation with triglyceride-OOH levels (β = −0.02, *t* = −2.04, *p* = 0.046), since HDL-C levels explain 5% of the variance [*R*^2^ = 0.05, *F*(1,62) = 4.16, *p* = 0.046] (Figure 7D). Similarly, there is statistically significant inverse correlation of the HDL-C with the levels of free cholesterol-OOH (β = −0.01, *t* = −3.05, *p* = 0.003), since HDL-C levels explain 12% of the variance [*R*^2^ = 0.12, *F*(1,62) = 9.28, *p* = 0.003] (Figure 7C).

**FIGURE 8:**
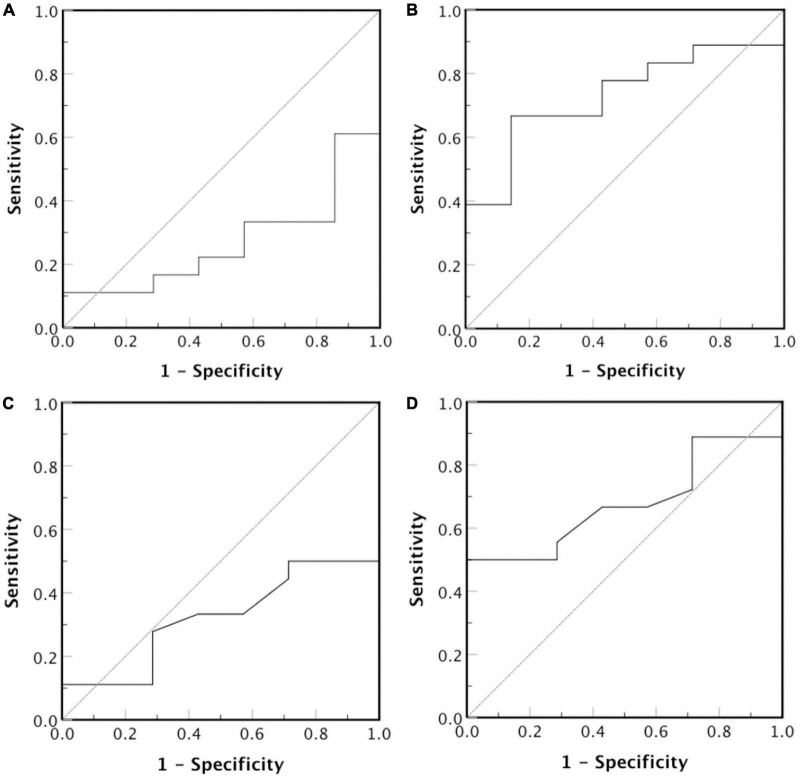
Receiver operator characteristic curve for LDL-P diameter (nm) and serum concentration (nmoles/L). The test hypothesis is if **(A)** smaller or **(B)** larger LDL-P particles, and **(C)** smaller or **(D)** larger concentration of LDL-P particles correlate with high risk for CVDs development.

We also investigated, using *ROC analysis*, whether LDL-P size correlates with LDL-C in order to serve as predictor for CVDs risk development in CKD-5d patients. Such analysis shows that smaller LDL-P size do not predict higher risk [Figure 8; area under curve (AUC) = 0.27, 95% confidence interval (CI): 0.07–0.47, *p* = 0.08], whereas larger LDL-P size is a statistically insignificant CVDs risk predictor (Figure 8; AUC = 0.73, 95% CI: 0.53–0.93, *p* = 0.08). Regarding LDL-P serum concentration as predictor for CVDs risk development, the ROC analysis shows that higher concentration of LDL-P is statistically insignificant (Figure 8; AUC = 0.68, 95% CI: 0.47–0.89, *p* = 0.11), whereas smaller LDL-P concentrations do not predict higher CVDs risk (Figure 8; AUC = 0.32, 95% CI: 0.11–0.53, *p* = 0.11).

## 5. Discussion

The present study uses clinically applicable, simple, innovative methods (Figure 2) to isolate LDL, measure its purity, size and serum concentration, and fractionate it into apoB100, phospholipids, triglycerides, free cholesterol, and cholesteryl esters (together with carotenoids and tocopherols). Selected specific oxidative modifications in the aforementioned LDL protein/lipid sub-fractions, together with LDL-P size and serum concentration, are quantified as potential clinical markers of oxLDL status in CKD-5d patients and for possible association with CKD-5d CVDs risk assessment. The innovations of this study are:

• Development of a simple, relatively time-short, low-cost protocol for LDL isolation, to avoid shortcomings of the ultracentrifugation [up to 60 h long (24, 25, 59)] and affinity chromatography methodologies. The first method is also prone to contamination by other lipoproteins (26), while the second is based on apoB100 antibodies immobilized on crosslinked agarose (26), which may possibly not be able to retain those oxLDLs having their apoB100 antibody epitopes oxidatively modified in many ways by OS (Figure 1). Isolated LDL recovery is >90%, which is comparable to that of ultracentrifugation (32, 36).
• Development of a method for LDL-P size and serum concentration determination in contrast to the, specialized equipment requiring, NMR (53–55) and gradient gel electrophoresis methods (53, 60). NMR’s use for LDL-P size and concentration is restricted to clinical research due to the absence of detailed analytical procedures and the absence of calibration and validation procedures (53), whereas electrophoresis for LDL-P size is a time-demanding (20–24 h) and cumbersome method (53, 61).
• Development, for the first time, of simple protocols for the fractionation of LDL into its main protein/lipid/antioxidant components. Their application can be extended to other biological sources such as blood serum, organ tissues, plants, microorganisms, and cells.
• Measurement, for the first time, of specific LDL protein/lipid component oxidative modifications, selected on the basis of representing early OS-prone oxidative effects. These are applied on CKD-5d patients as indicative markers of oxLDL and for possible association with CKD-5d CVDs risk assessment. The selected markers are cholesteryl ester-OOH, triglyceride-OOH, free cholesterol-OOH, phospholipid-OOH, apoB100-MDA, and apoB100-DiTyr, and they partially express oxLDL status as being part of the many known (Figure 1) and unknown markers that collectively define oxLDL status. Our oxLDL status assessment approach based on the specific OS-induced markers of LDL components, contrasts with the current oxLDL non-specific evaluation methods, where oxLDL is measured as a whole particle indirectly and non-specifically by mainly two immunological methods: The first method measures the immunogenic response (generation of serum autoantibodies) against artificially produced oxLDL (from LDL existing in an ever-changing unspecified oxidative status across general population), whereas the second one uses murine monoclonal antibodies for measuring serum oxLDL (62, 63). Besides their non-specificity for oxLDL, both methods are non-standardized in terms of reproducibility (between different kits and different batches of the same kit), rendering them unreliable in comparisons among different clinical studies regarding oxLDL and CVDs development (10, 63–66); detailed reference to these and other problems is made elsewhere (63, 67).

The choice of the study group and the selected oxLDL status markers, representing early OS appearing oxidative modifications of LDL components, are justified as such because chronic kidney disease (CKD) patients exhibit high OS (28) and develop atherosclerotic lesions, already in the early stages of renal dysfunction (27), which are further aggravated by hemodialysis (28). The latter is in accordance with our finding that the levels of all markers tested on CKD-5d patients are not affected by the number of years of hemodialysis, something that is also in concert with previous findings suggesting that cardiovascular events appear in high frequency even in patients with ESRD that just started hemodialysis (68, 69). Moreover, ESRD patients present a prolonged LDL stay in blood circulation (30) that together with high OS levels could lead to oxLDL generation, which in turn may reflect the high atherosclerosis prevalence in this group. Nonetheless, the established clinical cardiovascular risk markers do not correlate with the cardiovascular outcome in CKD-5d patients (31, 68), raising the need for new more reliable clinical markers for CVDs risk assessment and as early as possible for this group of patients (68, 70). Although, oxLDL is a promising CVDs clinical marker, only a few studies have investigated the relationship between oxLDL and CVDs development in hemodialysis patients (71–78). However, all of these studies have used the aforementioned non-specific oxLDL measurement methods.

The selected in the present study oxLDL protein/lipid component markers are measured in CKD-5d patients (before and after hemodialysis) and compared to the control group. Only the lipid peroxidation related markers triglyceride-OOH and free cholesterol-OOH are elevated in CKD-5d patients compared to the control group, which signifies a possible causative association of oxidized cholesterol with CKD-5d. It is very interesting to note also that the increased levels of the aforementioned markers are not associated with patients’ underlying medical conditions, suggesting that CKD can be related mostly to high OS. This is also corroborated by the fact that not even statin medication correlates with the two OS markers in CKD-5d patients, which is in accordance with the observation of no beneficial effect by statins in any underlying CVD of these patients (79–81). This is corroborated also with our finding that LDL-C levels in CKD-5d patients correlate inversely with free cholesterol-OOH levels, and is also consistent with the finding that patients with advanced CKD and low LDL-C levels present higher risk for all cause death (16). All these findings further strengthen the disputed role of LDL-C as a reliable universal marker for CVDs development (12, 13, 15). In contrast, our study shows that HDL-C levels in CKD-5d patients correlate inversely with LDL triglyceride-OOH and free cholesterol-OOH. This supports the reported inverse relationship between HDL-C levels and the occurrence of cardiovascular events (82), and is also consistent with a previous study where HDL-C in CKD-5d patients was found to be also inversely correlated with oxLDL levels, although defined by non-specific methodology (77). Finally, it is found that LDL-C levels do not correlate neither with LDL-P size nor LDL-P concentration in CKD-5d patients, and support the unclear clinical role of these parameters as predictors for CVDs risk (83).

## 6. Conclusion

By isolating LDL and fractionating LDL’s protein and main lipid components, as well as its antioxidant arsenal comprised of carotenoids and tocopherols, the present study paves the way for future studies to investigate additional oxidative modifications that complement the markers that define oxLDL status, thus its more reliable association with CVDs risk assessment. Moreover, the separate fractions of carotenoids and tocopherols isolated by the present study, facilitate the identification of their individual species (57, 58), and promote further studies on their association with LDL oxidation and atherogenesis (58, 84).

## Data availability statement

The raw data supporting the conclusions of this article will be made available by the authors, without undue reservation.

## Ethics statement

The studies involving human participants were reviewed and approved by the Scientific Committee of the General University Hospital of Patras. The patients/participants provided their written informed consent to participate in this study.

## Author contributions

PP: methodology, investigation, formal analysis, data visualization, statistical analysis, and writing—original draft, and review and editing. MSk, EK, AV, and MSp: investigation, formal analysis, and writing—review and editing. MP, EP, and DG: clinical study design on chronic kidney disease patients and review and editing. AO: investigation. ER and IM: writing—review and editing. CG: conceptualization, methodology, validation, formal analysis, graphical data/graphical visualization, supervision, writing—original draft and review and editing, and project administration. All authors contributed to the article and approved the submitted version.
