# Supplementary material for: Methods on LDL particle isolation, characterization, and component fractionation for the development of novel specific oxidized LDL status markers for atherosclerotic disease risk assessment
Source: Front Med (Lausanne). 2023 Jan 5;9:1078492. doi: 10.3389/fmed.2022.1078492 (PMC9851470; doi:10.3389/fmed.2022.1078492)
Supplement: Supplementary file 1 [file Data_Sheet_2.PDF]

Supplementary Material

I. Subjects

The demographic and clinical data for all 61 CKD-5d patients participating in the present clinical study are presented in the following **Table S1**.

**Table S1: CKD-5d patient age, sex and medical status**		
**Medical status**		**N**
**Age**	20 to 94	
Mean (±SD)	61.9 (±15.2)	
**Sex**		
Male		41
Female		20
**Years of hemodialysis**	0.5 to 24.2	
Mean (±SD)	6.4 (± 4.6)	
**Medical conditions**		
Hypertension		26
Coronary heart disease (CHD)		11
Peripheral artery disease (PAD)		24
Cardiovascular disease (CVD)		34
Diabetes		19
**Medication**		
Statin treatment		22
Alfacalcidol		8
Paricalcitol		11
ACE inhibitors		3
Angiotensin receptor blockers (ARBs)		11
Calcium channel blockers (CaChBI)		15
Beta blockers		35
Levocarnitine		15
Vitamin B supplement		19

II. LDL cholesteryl ester and triglyceride sub-fractions identification by TLC

Cholesteryl ester and triglyceride TLC zones (fractionated in *sub-section* **3.3**, **steps 2, 3**) are identified by comparison of their Rfs with those already reported in the literature for the same lipid fractions **(1)**. Before sample loading, TLC aluminum silica gel 60 sheets (5x5 cm) are previously dried at 60°C in an oven for 1 hour. Lipid sub-fraction samples (~2 μl) are loaded on the pretreated TLC plates, and exposed to 2.5% diethyl ether in benzene (the developing solvent is the same as used by **(1)**) followed by air-drying at 60°C, and then immersed in a beaker containing 0.005% Rhodamin 6G solution for band staining, followed by plate air-drying and band visualization by a UV-C lamp at 254 nm (**Fig. S1**). The Rf for each lipid fraction is calculated as follows: Rf = distance traveled by lipid compound/distance traveled by solvent front. Rfs calculated by our TLC are: $Rf_{\text{cholesteryl esters}} = 0.94$, $Rf_{\text{Triglycerides}} = 0.73$, which match with the Rfs calculated from **(1)** ($Rf_{\text{cholesteryl esters}} = 0.96$, $Rf_{\text{Triglycerides}} =$

0.71). The aforementioned results confirm that our isolated fractions are indeed cholerestyl esters and triglycerides.

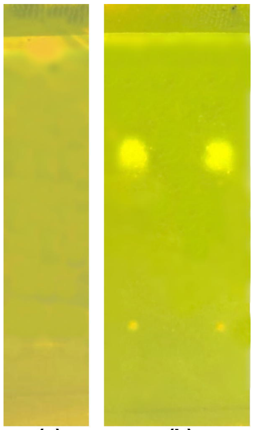

Figure S1: TLC of (a) LDL cholesteryl ester sub-fraction and (b) LDL triglyceride sub-fraction.

III. Measurement of specific oxidative modifications in LDL's main components in CKD-5d patients before and after hemodialysis

It is investigated whether one hemodialysis session has an effect on all six oxLDL-associated specific markers (cholesteryl ester-OOH, triglyceride-OOH, free cholesterol-OOH, phospholipid-OOH, apoB100-MDA, and apoB100-DiTyr) measured in LDL sub-fractions of CKD-5d patients (**Table S2**).

Table S2: Oxidation markers of LDL lipid sub-fractions and apoB100 in CKD-5d patients before and after hemodialysis

Oxidative marker	Subjects	Value	p-value
Cholesteryl ester-OOH	CKD5d-BH	0.95 (±0.76)	0.23
	CKD5d-AH	1.10 (±1.29)	
Triglyceride-OOH	CKD5d-BH	1.52 (±1.04)	0.87
	CKD5d-AH	1.59 (±1.27)	
Free cholesterol-OOH	CKD5d-BH	0.40 (±0.35)	0.85
	CKD5d-AH	0.39 (±0.40)	
Phospholipid-OOH	CKD5d-BH	0.18 (±0.19)	0.93
	CKD5d-AH	0.24 (±0.47)	
apoB100-MDA	CKD5d-BH	15.68 (± 5.97)	0.48
	CKD5d-AH	16.09 (±5.44)	
apoB100-DiTyr	CKD5d-BH	10.85 (± 5.41)	0.07
	CKD5d-AH	9.86 (± 5.11)	

Table notes:
Values are presented as mean (M) and standard deviation (SD).
Before and after hemodialysis serum testing is designated CKD5d-BH and CKD5d-AH, respectively.
LDL sub-fraction-OOH marker is expressed as cum-OOH nmole equivalents/mg apoB100, apoB100-MDA marker as MDA pmole /mg apoB100, and apoB100-DiTyr marker as DiTyr pmole /mg apoB100.

IV. OxLDL-measured specific oxidative modifications in CKD-5d patients in reference to their sex and medical conditions

Table S3: Oxidation markers of LDL free cholesterol and triglyceride sub-fraction in CKD-5d patients before hemodialysis according to sex and medical conditions					
	**LDL oxidation markers**	**CKD-5d patients**	**N**	**Oxidative value**	**p-value**
**Sex**	Free cholesterol-OOH	female	20	0.32 (±0.27)	0.23
		male	41	0.44 (±0.38)	
	Triglyceride-OOH	females	20	1.18 (±0.71)	0.06
		males	41	1.68 (±1.14)	
**Hypertension**	Free cholesterol-OOH	with hypertension	26	0.36 (±0.26)	0.61
		no hypertension	35	0.44 (±0.40)	
	Triglyceride-OOH	with hypertension	26	1.29 (±0.70)	0.29
		no hypertension	35	1.69 (±1.22)	
**PAD**	Free cholesterol-OOH	with PAD	24	0.42 (±0.41)	0.88
		no PAD	37	0.39 (±0.31)	
	Triglyceride-OOH	with PAD	24	1.69 (±1.23)	0.47
		no PAD	37	1.41 (±0.89)	
**CHD**	Free cholesterol-OOH	with CHD	11	0.36 (±0.27)	0.85
		no CHD	50	0.41 (±0.36)	
	Triglyceride-OOH	with CHD	11	1.61 (±1.12)	0.73
		no CHD	50	1.50 (±1.04)	
**CVD**	Free cholesterol-OOH	with CVD	34	0.43 (±0.43)	0.93
		no CVD	27	0.36 (±0.23)	
	Triglyceride-OOH	with CVD	34	1.67 (±1.24)	0.54
		no CVD	27	1.34 (±0.73)	
**Diabetes**	Free cholesterol-OOH	with diabetes	19	0.42 (±0.46)	0.63
		no diabetes	42	0.39 (±0.29)	
	Triglyceride-OOH	with diabetes	19	1.38 (±1.04)	0.35
		no diabetes	42	1.58 (±1.04)	

Table notes:
Values are presented as mean (M) and standard deviation (SD).
Free cholesterol-OOH and triglyceride-OOH marker is expressed as cum-OOH nmole equivalents/mg apoB100.

V. OxLDL-measured specific oxidative modifications in CKD-5d patients in reference to their medication

Table S4: Oxidation markers of LDL free cholesterol and triglyceride sub-fraction in CKD-5d patients before hemodialysis according to their medication					
**Medication**	**LDL oxidation markers**		**N**	**Oxidative value**	**p-value**
**Alfacalcidol**	Free cholesterol-OOH	TM	8	0.37 (± 0.22)	0.94
		NM	53	0.41 (± 0.37)	
	Triglyceride-OOH	TM	8	1.53 (± 0.95)	0.88

		NM	53	1.52 (± 1.06)	
**Vitamin B**	Free cholesterol-OOH	TM	20	0.45 (± 0.43)	0.53
		NM	41	0.38 (± 0.30)	
	Triglyceride-OOH	TM	20	1.57 (± 1.03)	0.71
		NM	41	1.49 (± 1.06)	
**Paricalcitol**	Free cholesterol-OOH	TM	11	0.42 (± 0.33)	0.77
		NM	50	0.39 (±0.36)	
	Triglyceride-OOH	TM	11	1.58 (±0.82)	0.62
		NM	50	1.57 (±1.12)	
**ACE inhibitors**	Free cholesterol-OOH	TM	3	0.53 (±0.22)	0.22
		NM	58	0.39 (± 0.35)	
	Triglyceride-OOH	TM	3	1.15 (± 0.81)	0.62
		NM	58	1.54 (± 1.05)	
**ARBs**	Free cholesterol-OOH	TM	11	0.30 (± 0.18)	0.48
		NM	50	0.42 (± 0.37)	
	Triglyceride-OOH	TM	11	1.48 (± 1.09)	0.74
		NM	50	1.55 (± 1.09)	
**CaChBI**	Free cholesterol-OOH	TM	15	0.32 (± 0.24)	0.32
		NM	46	0.43 (± 0.38)	
	Triglyceride-OOH	TM	15	1.14 (± 0.64)	0.10
		NM	46	1.64 (± 1.12)	
**Beta blockers**	Free cholesterol-OOH	TM	35	0.44 (± 0.32)	0.12
		NM	26	0.34 (± 0.39)	
	Triglyceride-OOH	TM	35	1.56 (± 1.08)	0.77
		NM	26	1.47 (± 1.01)	
**Levocarnitine**	Free cholesterol-OOH	TM	15	0.42 (± 0.26)	0.28
		NM	46	0.39 (± 0.38)	
	Triglyceride-OOH	TM	15	1.20 (± 0.67)	0.16
		NM	46	1.62 (± 1.12)	

Table notes:
CKD-5d patients taking/not taking the corresponding medication (TM/NM).
Values are presented as mean (M) and standard deviation (SD).
Free cholesterol-OOH and triglyceride-OOH markers are expressed as cum-OOH nmole equivalents/mg apoB100.

References

1. Tattrie HN. Isolation and identification of egg yolk cholesteryl esters. Can J Biochem (1972) 50:1966-75.
